# A single amino acid substitution in the AAA-type ATPase LRD6-6 activates immune responses but decreases grain quality in rice

**DOI:** 10.3389/fpls.2024.1451897

**Published:** 2024-08-06

**Authors:** Junjie Yin, Cheng Zhang, Qianyu Zhang, Feiyan Long, Wen Hu, Yi Zhou, Fengying Mou, Yufeng Zhong, Bingxiu Wu, Min Zhu, Lijuan Zou, Xiaobo Zhu

**Affiliations:** ^1^ State Key Laboratory of Crop Gene Exploration and Utilization in Southwest China, Sichuan Agricultural University at Wenjiang, Chengdu, Sichuan, China; ^2^ Agricultural and Rural Bureau, Hanyuan County Government, Yaan, Sichuan, China; ^3^ Ecological Security and Protection Key Laboratory of Sichuan Province, Mianyang Teachers’ College, Mianyang, China

**Keywords:** AAA-type ATPase, multivesicular bodies, vesicle trafficking, immune response, grain quality, rice

## Abstract

Plant *spotted leaf* (*spl*) mutants are useful to reveal the regulatory mechanisms of immune responses. Thus, in crop plants, their agronomic traits, especially the grain quality are usually ignored. Here, we characterized a rice *spl* mutant named *spl-A* (*spotted leaf mutant from A814*) that shows autoimmunity, broad-spectrum disease resistance and growth deterioration including decreased rice quality. A single nucleotide mutation of C1144T, which leads to change of the 382^nd^ proline to serine, in the gene encoding the ATPases associated with diverse cellular activities (AAA)-type ATPase LRD6-6 is responsible for the phenotype of the *spl-A* mutant. Mechanistically, this mutation impairs LRD6-6 ATPase activity and disrupts its interaction with endosomal sorting complex required for transport (ESCRT)-III subunits OsSNF7.1/7.2/7.3. And thus, leading to compromise of multivesicular bodies (MVBs)-mediated vesicle trafficking and accumulation of ubiquitinated proteins in both leaves and seeds of *spl-A*. Therefore, the immune response of *spl-A* is activated, and the growth and grain quality are deteriorated. Our study identifies a new amino acid residue that important for LRD6-6 and provides new insight into our understanding of how MVBs-mediated vesicle trafficking regulates plant immunity and growth, including grain quality in rice.

## Introduction

During the long-term of plant-pathogen interaction, plants have evolved with a layered immune system to detect and fight with various of pathogens. Pattern recognition receptors (PRRs) localized at the plasm membrane function as the first layer to detect pathogen derived molecular patterns and activate pattern-triggered immunity (PTI) (Jones and Dangl, 2006; Zhao et al., 2022). Intracellular receptors such as nucleotide-binding, leucine-rich repeat receptors (NLRs) function as the second layer to monitor pathogen secreted effectors to activate the more potent immune responses, effector-triggered immunity (ETI) (Jones and Dangl, 2006; Zhao et al., 2022). Once the immune responses are activated, plant will increase the expression of *pathogenesis-related* (*PR*) genes, induce the production of reactive oxygen species (ROS) and promote the synthesis of antimicrobial metabolites etc (Tsuda and Katagiri, 2010; Yuan et al., 2021). Finally, the immunity system may launch hypersensitive response (HR) to trigger cell death in and around the infection sites to restrict pathogen invasion and prevent disease development (Mur et al., 2008; Balint-Kurti, 2019).

It’s interesting that in the absence of pathogen infection, insect feeding, stress or mechanical damages, many plant mutants have been found to exhibit spontaneous, HR-like cell death spots on their bodies (Zhu et al., 2020). These mutants are named based on their phenotype as *spotted leaf* (*spl*), *lesion mimic mutant* (*lmm*), *accelerated cell death* (*acd*) and others (Bruggeman et al., 2015). Since these *spl* mutants usually carry the characteristics of activated immune responses, they are useful to uncover the regulatory mechanisms of plant immune responses. More than 35 genes that responsible for *spl* phenotype have been identified in rice at present (Zhu et al., 2020; Jiang et al., 2022; Ren et al., 2022; Zheng et al., 2022; Wang et al., 2023). Studies on these genes have revealed many processes or pathways are involved in plant immune responses regulation. These include the gene transcription and protein translation process, post-translational protein modification, the vesicle trafficking pathways, and several cellular metabolic pathways (Zhu et al., 2020). During the study of these genes or mutants, it has also found that these genes are usually involved in the regulation of plant growth as well (Zhu et al., 2020). However, little attention has been paid for their roles in plant growth.

The vesicle trafficking apparatus are critical for the growth and development of eukaryotes. Up to now, at least five vesicle-mediated trafficking pathways have been identified in plant. The coat protein I (COPI)-coated vesicles and the COPII-coated vesicles are involved in endoplasmic reticulum (ER)-Golgi trafficking, the clathrin-coated vesicles (CCVs) mediates endocytosis and post-Golgi trafficking, the exocyst positive organelles (EXPO) regulates exocytosis, and the multivesicular bodies (MVBs) modulates endocytosis, exocytosis, recycling and degradation of cargoes (Aniento et al., 2021; Zhu et al., 2023).

By studying of the *spl* mutants, several vesicle trafficking pathways have been found to be essential for rice immunity. SPL35 is a CUE domain-containing protein that interacts with the coatomer subunits δ-COP1 and δ-COP2 of the COPI vesicle (Ma et al., 2019). Suppression any of the three genes will lead to *spl* phenotype (Ma et al., 2019), providing evidence that the COPI trafficking pathway is involved in the regulation of immunity and HR-like cell death in rice. Loss of function of the *trans*-Golgi network (TGN)-derived CCVs components, SPL28, OsSCYL2 or OsCHC1 results in autoimmunity and cell death of rice (Qiao et al., 2010; Yao et al., 2022). OsSEC3A is a core subunit of the exocyst complex. Knockout of *OsSEC3A* causes enhanced immune responses and HR-like cell death likely due to the disruption of the EXPO-mediated exocytosis (Ma et al., 2017). The AAA-type ATPase LRD6-6 is a rice homology of VPS4/SKD1 which crucial for MVBs-mediated trafficking of ubiquitinated plasm membrane proteins (Zhu et al., 2016). During the formation of the MVBs, the ESCRT-I, -II, and -III complexes are sequentially recruited to coordinately drive vesicle maturation. At the final step, the VPS4/SKD1 assembles with the ESCRT-III at the neck region of the limiting membrane to hydrolyzes ATP and thus provide energy to facilitate neck scission and disassembly of the ESCRT-III complex (Piper and Katzmann, 2007; Hurley, 2008; Schmidt and Teis, 2012). Mutation of *Lrd6-6* in rice leads to autoimmunity and HR-like cell death (Zhu et al., 2016), indicating the important roles of LRD6-6 and MVBs-mediated trafficking in rice immune responses.

Besides their roles in plant immunity, the vesicle trafficking pathways have also been found to regulate rice chalkiness through regulating the transportation of storage proteins in endosperm, thus contributing to grain quality formation (Zhu et al., 2023). Rice endosperm cells contain two types of protein bodies, protein body I (PBI) and PBII, which are responsible for protein storage in seed. PBIs storage prolamins while PBIIs mainly accumulate glutelins and globulins (Li et al., 2022). The delivery of the storage proteins into PBIs and PBIIs are tightly regulated by vesicle trafficking. This is mainly achieved by studying of *glutelin precursor accumulation* (*gpa*) mutants with abnormal endosperm and decreased grain quality in rice (Li et al., 2022; Zhu et al., 2023). Interesting, loss of function of an ESCRT-II complex subunit OsVPS22 also leads to chalky endosperm likely for impaired grain filling (Zhang et al., 2013), suggesting that the MVBs-mediated vesicle trafficking pathway may also likely involved in storage protein transportation in rice endosperm cells. However, our knowledges about whether and how MVBs-mediated vesicle trafficking regulate rice endosperm development and grain quality formation are still remain largely unknown.

In this study, we isolated and characterized the rice spotted leaf mutant *spl-A*, which shows autoimmunity, broad-spectrum disease resistance and growth deterioration including decreased rice quality. By combining map-based cloning strategy and bulked segregant analysis (BSA) sequencing, we found a single nucleotide mutation of C1144T in the gene encoding the AAA-type ATPase LRD6-6 is responsible for the phenotype of the *spl-A* mutant. This mutation leads to change of the 382^nd^ proline to serine, impairs LRD6-6 ATPase activity and disrupts its interaction with ESCRT-III subunits OsSNF7.1/7.2/7.3. And finally, result in compromise of MVBs-mediated vesicle trafficking and accumulation of ubiquitinated proteins in *spl-A*. Collectively, our results identify a new amino acid residue that important for LRD6-6 and point to a novel function for MVBs-mediated vesicle trafficking regulates grain quality in rice.

## Materials and methods

### Plant materials and growth conditions

The *spl-A* mutant was isolated from the methanesulfonate (EMS) treated *japonica* rice A814 (*XA21*-*OsSERK2Ri* in Kitaake) (Caddell et al., 2017). Two F_2_ populations derived from the cross Jodan×*spl-A* and *spl-A*×Jodan were used for genetic analyses and mapping of the *spl-A* locus. All plants for mapping were grown in the controlled fields at Sichuan Agricultural University in Wenjiang, Chengdu, China.

For major agronomic traits analysis, plant materials were cultivated in the controlled fields with 6 rows (12 plants per row) at Wenjiang district, Chengdu, China. At maturity, forty plants in the middle row were selected for plant height, tiller number and panicle length determination, and then they were harvested respectively and air-dried. For seed setting rate and 1,000-grain weight, five A814 and *spl-A* plants were randomly and respectively selected. Scanning electron microscopy observation was performed as described before (Ren et al., 2020). For grain quality determination, the seeds from all the forty plants of A814 and *spl-A* were mixed respectively, and chalky grain percentage and chalkiness degree were detected using the WSeen SC-E type rice appearance quality detection analyzer (Wanshen Detection, Hangzhou, China) with three technical repetitions. Student’s *t*-test was used to compare the differences between A814 and *spl-A* and the data were analyzed by using Microsoft Excel 2019.

### Histochemical analysis

Trypan blue staining and DAB (3, 3’-diamiobenzidine) staining were performed on fresh leaves following the method as described previously (Zhu et al., 2016). Briefly, for trypan blue staining, samples were submerged in lactic acid-phenol-trypan blue solution (2.5 mg/ml trypan blue, 25% (w/v) lactic acid, 23% water-saturated phenol and 25% glycerol in H_2_O) and were boiled in water for 2 min, then de-stained with solution containing 30% (w/v) chloral hydrate for 3 days with multiple exchanges of the solution. After distained completely, the samples were then equilibrated with 50% glycerol for five hours followed by photo-picture taken. For DAB staining, leaf samples were immersed in 1 mg/ml DAB containing10 mM MES (pH 6.5) for 12 h in the dark at 30°C. Then the leaf samples were transferred to solution containing 90% ethanol and 10% glycerol at 90°C until chlorophyll was completely removed. Images were acquired by a microscope (Zeiss imager A2).

### RNA isolation and RT-qPCR

For determination of gene expression levels, the desired samples were harvested and total mRNA were respectively extracted using TRIzol agent (15596018CN, Invitrogen, Carlsbad, USA) following the procedures described by the manufacturer. The mRNA was then subject to reverse transcription to synthesize first-strand cDNA by using the PrimeScript™ RT reagent Kit with gDNA Eraser (RR047A, Takara, Beijing, China) for genomic DNA removal.

The reverse transcription-quantitative PCR (RT-qPCR) was conducted using a Bio-Rad CFX96 Real-Time System coupled to a C1000 Thermal Cycler (Bio-Rad, Hercules, USA). The reference gene *Ubiquitin* (*Ubq*) was used as reference (Zhu et al., 2016). Primer sequences are listed in **Supplementary Table 1**.

### Disease resistance evaluation

For blast resistance, rice leaves from three-week-old seedlings of A814 and *spl-A* plants were used. The inoculation was performed as described by Li et al (Li et al., 2020). Briefly, the spore concentration of the blast was adjusted to 5×10^5^ conidia mL^-1^. 4 μL of the spore suspension was dipped onto the leaves with three spots per leaf. Then the leaves were kept in a culture dish contain 0.1% 6-benzylaminopurin (6-BA) for 5 days. The lesion length was measured and the relative fungi biomass was detected and calculated according to the method reported before (Li et al., 2020). The fungal isolates Zhong10-8-14, ZE-1, 0755-1-1 and 99-20-2 that are compatible with A814 were respectively used for blast resistance evaluation.

For bacterial blight disease, the *Xoo* strains P2, P6 and Xoo-4, which are compatible with A814 were used for inoculation. *Xoo* bacterial suspensions of OD600 = 0.5 were used to inoculate by using the scissors-dip method as described previously (Zhu et al., 2016). The lesion lengths and bacterial populations were determined respectively at day 0, 7, 14 post inoculation.

### Genetic analysis and cloning of the locus *Spl-A*

Two F_2_ populations derived from the cross Jodan×*spl-A* and *spl-A*×Jodan were used for genetic analyses and mapping of the *spl-A* locus as described before (Zhu et al., 2016). The SSR primers were synthesized according to the information from Gramene database (http://www.gramene.org/microsat).

For BSA sequencing, leaf samples of 30 F_2_ individuals with the *spl-A* phenotype and the wild-type phenotype were respectively mixed at an equal ratio and subjected to whole-genome resequencing and analysis at Biomarker Technologies Corporation (Beijing, China).

For candidate gene validation, the DNA segments containing the mutation sites were amplified using gene-specific primers and sequenced at Tsingke Biotech (Chengdu, China). The genotypes of the *lrd6-6*/*spl-A* F_1_ plants were also confirmed by sequencing of *spl-A* and *lrd6-6*-specific PCR-based agarose gel electrophoresis respectively. All the primers used for gene mapping and genotyping are listed in **Supplementary Table 1**.

### Plasmid construction

The desired DNA segments were amplified by using the Phanta Super-Fidelity DNA Polymerase (P505-d1, Vazyme Biotech, Nanjing, China). The segments were then inserted into the vectors to produce the final plasmids by using the ClonExpress II One Step Cloning Kit (C112-02, Vazyme Biotech, Nanjing, China). All the plasmids were then confirmed by sequencing at Tsingke Biotech (Chengdu, China) subsequently. The primers and vectors used were listed in **Supplementary Table 1**.

### ATPase activity assay

ATPase activity determination assay was performed as described previously (Zhu et al., 2016). The truncated protein His-LRD6-6(125-487)^P382S^ (covering the ATPase domain) was purified and subjected to this analysis. His-LRD6-6(125-487) and His-LRD6-6(125-487)^E315Q^ were included as positive and negative control respectively. ATPase activity of the recombinant proteins were measured by the malachite green-based colorimetric method (Zhu et al., 2016). The relative ATPase activity was calculated by setting the activity of His-LRD6-6(125-487) as 100%.

### Protein interaction detection and subcellular localization

Yeast two-hybrid (Y2H) assay and bimolecular fluorescence complementation (BiFC) were performed as described previously (Zhu et al., 2016).

For subcellular localization, plasmids were introduced into *Nicotiana benthamiana* leaf by agroinfiltration. Fluorescence was examined under a confocal microscopy (Leica STELLARIS STED, Germany) 48 h post transformation. The fluorescence intensity were measured by using ImageJ (National Institutes of Health, USA).

### Protein extraction and immunoblot analyses

For protein extraction from plant leaf samples, the denaturing buffer (50 mM Tris-HCl pH=7.5, 150 mM NaCl, 0.1% NP-40, 4 M urea and 1 mM PMSF) was used (Liu et al., 2010). For the dry seeds, the extraction buffer (125 mM Tris-HCl pH=6.8, 4% SDS, 4 M urea and β-mercaptoethanol) described before was used (Pan et al., 2021). The total protein content was determined by the Protein Content Assay Kit (BC3185, Solarbio, Beijing, China).

The proteins were resolved by sodium dodecyl sulfate polyacrylamide gel electrophoresis (SDS-PAGE) analysis and followed by Coomassie brilliant blue staining or electro transfer to nitrocellulose membranes to perform immunoblot analyses using anti-His antibody (ab18184, Abcam, Shanghai, China), anti-Ub (P4D1) antibody (ab303664, Abcam, Shanghai, China) and anti-HSP82 antibody (AbM51099-31-PU, Beijing Protein Innovation, Beijing, China).

## Results

### Characterization of the *spl-A* mutant in rice

**Figure 1:**
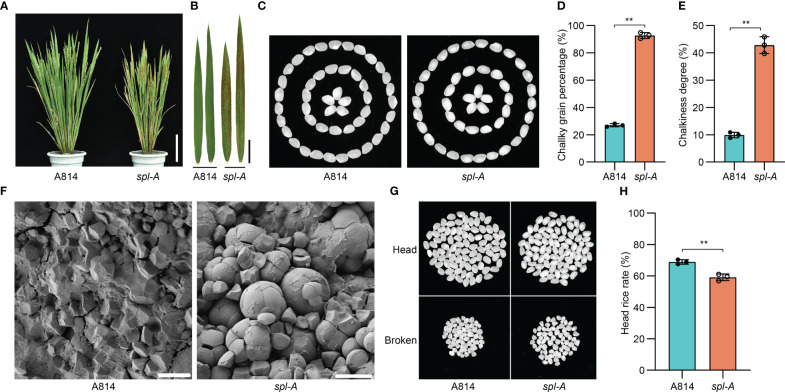
Phenotypic characterization of the *spl-A* mutant. **(A, B)** Photographs of the wild-type A814 and the *spl-A* mutant plants **(A)**, and their leaves **(B)** at the filling stage. Bar = 10 cm in **(A)**, Bar = 2 cm **(B)**. **(C)** The grain appearance of A814 and *spl-A*. **(D, E)** Comparison on the chalky grain percentage **(D)** and chalky grain degree **(E)** between A814 and *spl-A* (mean ± s.d.). **(F)** Scanning electron microscopy images of transverse sections on the wild-type A814 and the *spl-A* mutant dry seeds. Bars = 10 μm. **(G, H)** The head rice rate of the *spl-A* mutant. Representative images of 100 dehulled and milled rice grains per genotype were separated into head and broken grains **(G)**. **(H)** Statistical comparison of head rice rate between A814 and *spl-A* seeds (mean ± s.d.). All statistics were analyzed by Student’s *t*-test for *P* values (**, *P* <= 0.01; *, *P* <= 0.05; ns, no significant differences).

The spotted leaf mutant which we named *spl-A* (*spotted leaf mutant from A814*) was isolated from ethyl methanesulfonate (EMS) treated *japonica* rice A814 for its visible lesion mimic phenotype on the leaves when growing in the field. The *spl-A* mutant exhibited reddish-brown lesion spots on the leaves about 20 days after sowing and the lesion spots expand through the entire plants along with development (**Figures 1A, B**). To examine whether the agronomic traits including grain quality were affected in *spl-A*, we compared the chalky grain percentage, chalky grain degree, plant height, tiller number, panicle length, seed setting rate, 1,000-grain weight, and grain size between A814 and *spl-A*. It was found that the chalky grain percentage and chalky grain degree were significantly increased (**Figures 1C–E**), indicating that the grain quality of *spl-A* was seriously deteriorated. The plant height, seed setting rate, 1,000-grain weight and grain length of *spl-A* were also decreased prominently when compared to those of the wild-type A814, while the tiller number, panicle length and grain width of *spl-A* were not affected (**Supplementary Figure 1**).

Grain chalkiness is the opaque part of translucent endosperm in grains that usually caused by altered package of the starch granules (Yao et al., 2020). We then analyzed the starch granules organization of *spl-A* seed. Scanning electron microscopy revealed that the *spl-A* mutant endosperm was composed of loosely arranged and round-shaped compound starch granules, while that of the wild-type A814 was tightly packed and polyhedral shaped (**Figure 1F**). Grain chalkiness often affects processing quality (Li et al., 2022). Indeed, the head rice rate of *spl-A* was prominently decreased when compared to A814 (**Figures 1G, H**). Taken together, these results indicate that *Spl-A* regulates multiple aspects of rice development including grain quality.

### The *spl-A* mutant presents autoimmunity and broad-spectrum disease resistance

**Figure 2:**
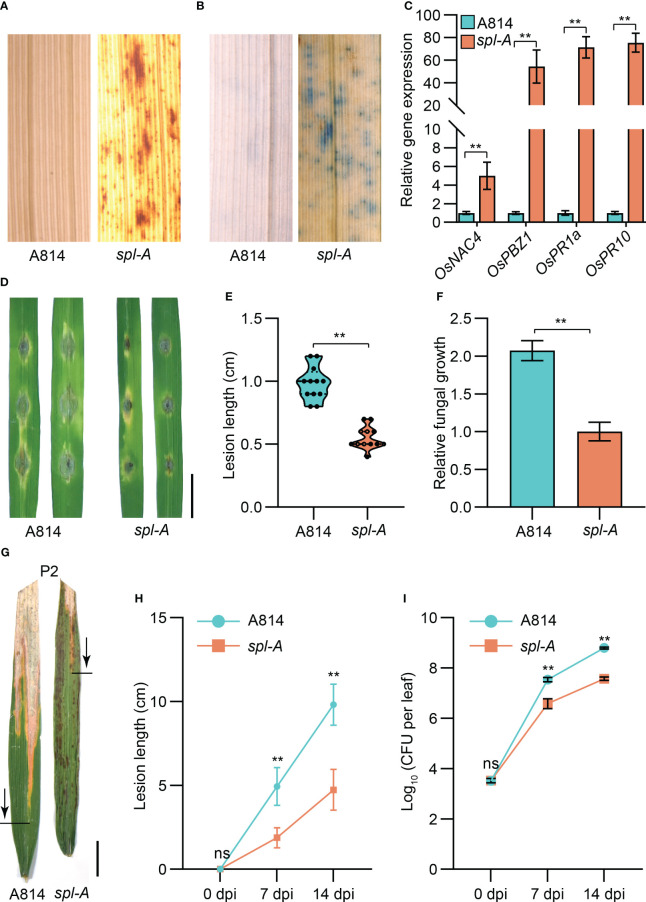
Determination on the immunity and disease resistance of the *spl-A* mutant. **(A, B)** The DAB staining **(A)** and trypan blue staining **(B)** analyses on A814 and *spl-A* at 15 days after sowing. **(C)** Comparison the expression levels of the *pathogenesis-related* (*PR*) genes between A814 and *spl-A* at 15 days after sowing. The expression levels of the *PR* genes were normalized to the reference gene *Ubiquitin* (mean ± s.d., n = 3 technical repetitions). **(D–F)** Evaluation on the blast resistance of the *spl-A* mutant. Punch inoculation method was employed. The blast fungal isolate Zhong10-8-14 that is compatible with A814 was used. Photograph of representative lesions **(D)** were shown. Bar = 1 cm. Lesion length **(E)** and the relative fungal growth **(F)** were measured at 5 day-post-inoculation (dpi). The relative fungal growth was determinated as fungi MoPot2 DNA to rice OsUbq DNA by qPCR (mean ± s.d., n = 3 technical repetitions). **(G–I)** Evaluation on the resistance of the *spl-A* mutant to bacterial blight disease. Photographs of representative leaves were taken at 14 dpi with *Xanthomonas oryzae pv oryzae* (*Xoo*) strain P2, which is compatible with A814 **(G)**. Bar = 1 cm. Disease lesion lengths **(H)** and bacterial populations **(I)** of the *spl-A* mutant and A814 were measured at 0, 7 and 14 dpi respectively (mean ± s.d., n = 12 for lesion lengths and n = 3 for bacterial populations). All statistics were analyzed by Student’s t-test for P values (**, P <= 0.01; ns, no significant differences).

The *spl* mutants usually exhibit excessive accumulation of hydrogen peroxide (H_2_O_2_) and autoimmunity which then lead to programmed cell death (Li et al., 2017; Yao et al., 2022). We thus employed the DAB (3, 3’-diamiobenzidine) staining and trypan blue staining methods to examine the accumulation of H_2_O_2_ and the existence of cell death in *spl-A* leaves respectively at 15 days after sowing, before visible lesions appeared. DAB staining analysis detected extensive stains in leaves of *spl-A* when compared to A814 (**Figure 2A**). Trypan blue staining also detected many blue staining spots on the leaves of *spl-A* (**Figure 2B**). To determinate whether the immune responses of the *spl-A* mutant was activated, we then compared the expression levels of immunity related genes, such as the *pathogenesis-related* (*PR*) genes, *OsNAC4*, *OsPBZ1*, *OsPR1a* and *OsPR10* between *spl-A* and A814 at 15 days after sowing (Li et al., 2017; Tao et al., 2021). We found that the expression levels of all the *PR* genes analyzed were largely increased in *spl-A* when compared to A814 (**Figure 2C**). These results suggest that the *spl-A* mutant accumulates an excess of H_2_O_2_ and high levels of *PR* genes which may cause cell death and enhance plant basal disease resistance.

We then challenged the *spl-A* mutant with the fungal pathogen *Magnaporthe oryzae* (*M. oryzae*) and the bacterial pathogen *Xanthomonas oryzae* pv *oryzae* (*Xoo*) which cause rice blast and bacterial blight diseases respectively. As expected, the lesion length and the relative fungal growth of leaves inoculated with *M. oryzae* strains (Zhong10-8-14, ZE-1, 0755-1-1 and 99-20-2) which compatible with A814 were all dramatically reduced in the *spl-A* mutant when compared with those of A814 (**Figures 2D–F** and **Supplementary Figure 2**). When inoculated with compatible *Xoo* strains (P2, P6 and Xoo-4), the *spl-A* mutant also exhibited enhanced bacterial resistance, showing much shorter lesion length and less bacteria than A814 (**Figures 2G–I** and **Supplementary Figure 3**). Collectively, these results indicate that the *spl-A* mutant possesses enhanced immunity and broad-spectrum disease resistance.

### Cloning of the *Spl-A* gene by combining map-based cloning strategy and BSA sequencing

**Figure 3:**
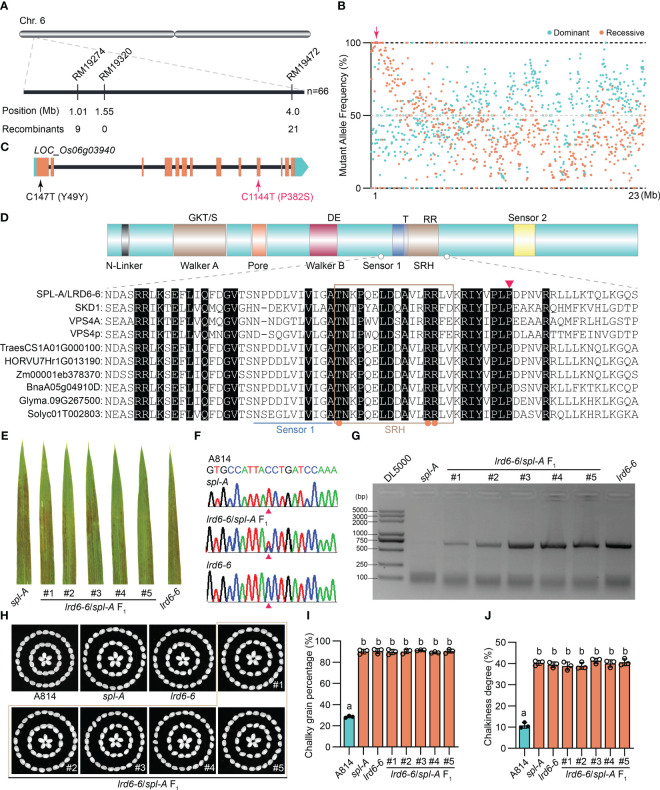
Molecular cloning and functional identification of *Spl-A* gene. **(A)** Preliminary mapping of the *Spl-A* locus. The *Spl-A* locus was delimited to a 3 Mb interval between SSR markers RM19274 and RM19472 on chromosome 6. The molecular markers and the number of recombinants is respectively shown. **(B)** Bulked segregant analysis (BSA) of the *Spl-A* locus. The candidate gene in the preliminary mapping interval is boxed and indicated by red arrow. **(C)** The schematic diagram of the *Spl-A* candidate gene identified by our combined strategy. The gene *LOC_Os06g03940* harbors two mutation sites in the *spl-A* mutant. One mutation is a nonsense mutation (C147T) in the first exon and another one is missense mutation (C1144T) in the 10^th^ exon that leads to change of Proline (P) to Serine (S). **(D)** Conserved structure of the LRD6-6 protein. The characteristics of typical AAA ATPase (upper panel) and partial sequence alignment analysis on LRD6-6 with some known AAA ATPases (lower panel) are respectively shown. The key elements of Walker A and B motifs, the Pore, Sensors 1 and 2, and the second region of homology (SRH) are respectively marked. The conserved residues in the SRH motif are indicated by orange dots. The 382^th^ amino acid P that mutated to S in *spl-A* is indicated by red triangle. **(E–J)** Allelic analysis between the *spl-A* and *lrd6-6* mutants. The leaves of *spl-A*, *lrd6-6* and five F_1_ progenies from the crossing of *lrd6-6* and *spl-A* were respectively shown **(A)**. The genotypes of the five F_1_ progenies were detected **(F, G)**. PCR-based sequencing was employed to determinate the C1144T mutant in *spl-A* **(F)**. The representative DNA sequencing chromatograms for the wild-type A814, the mutants *spl-A* and *lrd6-6*, and the F_1_ progenies are respectively shown. PCR-based agarose gel electrophoresis was used to distinguish the genotype of *lrd6-6*. The grain appearance of *spl-A*, *lrd6-6* and five F_1_ progenies from the crossing of *lrd6-6* and *spl-A* were also determined **(H–J)**. The photographs **(H)** and the chalky grain percentage **(I)**, chalky grain degree **(J)** are respectively shown. Statistical significance was determined by one-way ANOVA, where different letters indicate significant differences (*P* <= 0.01), while the same letter indicates no significant differences (*P* > 0.05).

In order to isolate the *Spl-A* gene, we reciprocally crossed the *indica* rice Jodan with the *spl-A* mutant and developed two F_2_ populations. Then, genetic analysis on the *spl-A* locus was carried out. We found the phenotype of *spl-A* was controlled by a single recessive nuclear locus through genetic analysis (**Supplementary Table 2**). Next, map-based cloning strategy and the bulked segregant analysis (BSA) sequencing method were simultaneously adopted to accelerate the cloning of *Spl-A* gene. The candidate gene was mapped in the interval with a physical distance of 3 Mb between the SSR markers RM19274 and RM19472 on chromosome 6 through map-based cloning strategy (**Figure 3A**). Combining with BSA sequencing analysis, we then identified the gene *LOC_Os06g03940* which harbored two single nucleotide mutations in the *spl-A* mutant was likely the candidate of *Spl-A* gene (**Figures 3B, C**). The C147T mutation in the first exon of *LOC_Os06g03940* is a nonsense mutation, while the C1144T mutation in the 10^th^ exon leads to change of 382^nd^ proline (P) to Serine (S) (**Figures 3B, C**).

The gene *LOC_Os06g03940* encoding an AAA-type ATPase has been previously reported by us as *Lesion resembling disease 6-6* (*Lrd6-6*). The *lrd6-6* mutant harbors a 1446-nt tandem repeat in *LOC_Os06g03940* gene, resulting in autoimmunity and broad-spectrum disease resistance (Zhu et al., 2016). We then found that the amino acid mutation P382S caused by the C1144T mutation in *spl-A* was conserved among the AAA-type ATPase family (**Figure 3D**). To further test whether *spl-A* and *lrd6-6* were allelic mutants caused by mutation of *LOC_Os06g03940*, we crossed *lrd6-6* with *spl-A* and investigated the phenotype of the F_1_ progenies. We found that all the F_1_ progenies from *lrd6-6*×*spl-A* exhibited lesion spots on the leaves as that of the *lrd6-6* and *spl-A* mutants (**Figure 3E**). The heterozygous genotypes (*spl-A*/*lrd6-6*) of the F_1_ progenies were confirmed by PCR-based sequencing and PCR-based agarose gel electrophoresis analysis respectively using specific primers (**Figures 3F, G** and **Supplementary Table 1**). Moreover, like the *lrd6-6* and *spl-A* mutants, the grain quality of all the F_1_ progenies were found to deteriorate as indicated by increased chalky grain percentage and chalky grain degree (**Figures 3H–J**). These results suggest that *spl-A* and *lrd6-6* are allelic mutants and the C1144T mutation in *LOC_Os06g03940* is responsible for the autoimmunity and decreased grain quality of the *spl-A* mutant. In order to maintain the consistency of the gene name, *Lrd6-6* was adopted to represent the *Spl-A* gene hereafter.

### The P382S mutation of LRD6-6 in *spl-A* impairs its ATPase activity and disrupts its interaction with ESCRT-III subunits OsSNF7.1/7.2/7.3

LRD6-6 is an active ATPase that can interact with itself and the ESCRT-III subunits OsSNF7.1/7.2/7.3 and OsVPS2 to regulate the MVBs-mediated vesicular trafficking pathway (Zhu et al., 2016). To determinate the effects of the P382S mutation in LRD6-6, we examined the ATPase activity of LRD6-6^P382S^, the interaction of LRD6-6^P382S^ with itself and the ESCRT-III subunits OsSNF7.1/7.2/7.3 and OsVPS2, and the MVBs localization of LRD6-6^P382S^.

**Figure 4:**
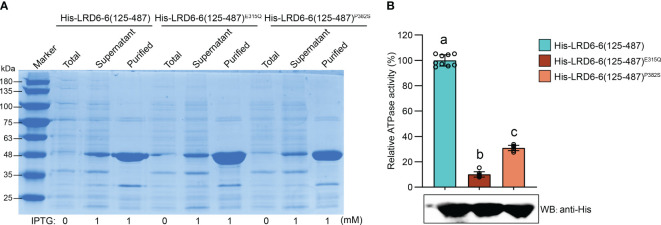
Determination of the ATPase activity on LRD6-6^P382S^ protein *in vitro*. **(A)** Expression and purification of the N-terminal truncated recombinant proteins His–LRD6-6(125–487), His–LRD6-6(125–487)^E315Q^ and His–LRD6-6(125–487)^P382S^ from *E. coli*. Coomassie brilliant blue staining was used to detect the total proteins from *E. coli* before IPTG addition, and proteins in supernatant and the purified proteins after IPTG induction. **(B)** *In vitro* ATPase assay on recombinant proteins His–LRD6-6(125–487), His–LRD6-6(125–487)^E315Q^ and His–LRD6-6(125–487)^P382S^. The proteins His–LRD6-6(125–487) and His–LRD6-6(125–487)^E315Q^ were used as positive and negative control respectively. ATPase activities were measured using a malachite green-based colorimetric approach (mean ± s.d., n = 9 technical repetitions). The purified proteins used for ATPase activity determination were detected by anti-His antibody. Statistical significance was determined by one-way ANOVA, where different letters indicate significant differences (*P* <= 0.01).

**Figure 5:**
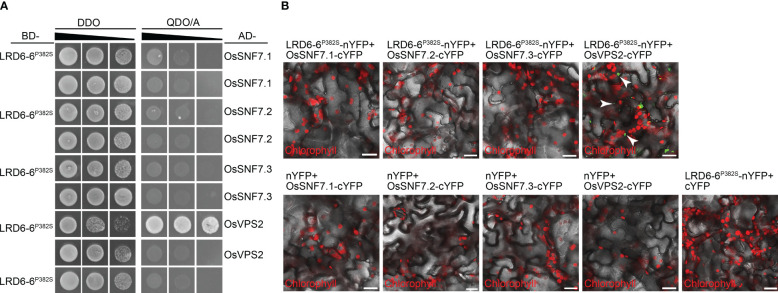
Detection the interaction between LRD6-6^P382S^ with OsSNF7 and OsVPS2. The interaction between LRD6-6^P382S^ with OsSNF7.1/7.2/7.3 and OsVPS2 were detected by using both yeast two hybrid (Y2H) **(A)** and bimolecular fluorescence complementation (BiFC) **(B)** approaches. BD, pGBKT7; AD, pGADT7; DDO, double dropout medium ((SD/–Leu/–Trp); QDO/A, quadruple dropout medium supplemented with Aureobasidin A (SD/–Ade/–His/–Leu/–Trp/AbA). BiFC assay was performed in *N. benthamiana*. The green fluorescence signals present the interaction between the tested proteins, the red signals represent the auto-fluorescence of chlorophyll. Some of the green fluorescence signals are indicated by white arrows to clearly shown the interaction. Bar = 20 μm.

We found that the P382S mutation in LRD6-6 impaired its ATPase activity because the His–LRD6-6(125–487)^P382S^ recombinant protein purified from *E. coli* showed significantly reduced ATPase activity when compared to the wild-type protein His–LRD6-6(125–487) (**Figure 4**). The self-interaction of LRD6-6 was not affected by the P382S mutation as detected by using the yeast two hybrid (Y2H) and bimolecular fluorescence complementation (BiFC) assays (**Supplementary Figure 4**). The interactions between LRD6-6^P382S^ and OsSNF7.1/7.2/7.3, LRD6-6^P382S^ and OsVPS2 were also examined using these two methods. And it was found that the interactions between LRD6-6^P382S^ and OsSNF7.1/7.2/7.3 were abolished while the interaction between LRD6-6^P382S^ and OsVPS2 was not affected (**Figure 5**). We also checked the MVBs localization of the protein LRD6-6^P382S^ by using RabF1/ARA6-RFP as the MVBs marker (Šamaj et al., 2005; Ebine et al., 2011). The co-localization of LRD6-6^P382S^-GFP punctate green fluorescence with RabF1/ARA6-RFP punctate red fluorescence was observed in *Nicotiana benthamiana* (*N. benthamiana*) cells, similar to that of the wild-type protein LRD6-6-GFP (**Supplementary Figure 5**), suggesting the P382S mutation in LRD6-6 not affects its MVBs localization.

Taken together, these results indicating that the conserved amino acid residue proline at 382^nd^ of LRD6-6 is essential for its ATPase activity and is also pivotal for interacting with the ESCRT-III subunits OsSNF7.1/7.2/7.3. These results further confirming that the C1144T mutation in *spl-A* which leads to P382S mutation of LRD6-6 is responsible for the autoimmunity, broad-spectrum disease resistance and decreased grain quality of the *spl-A* mutant.

### The MVBs-mediated vesicle trafficking is compromised in the *spl-A* mutant

**Figure 6:**
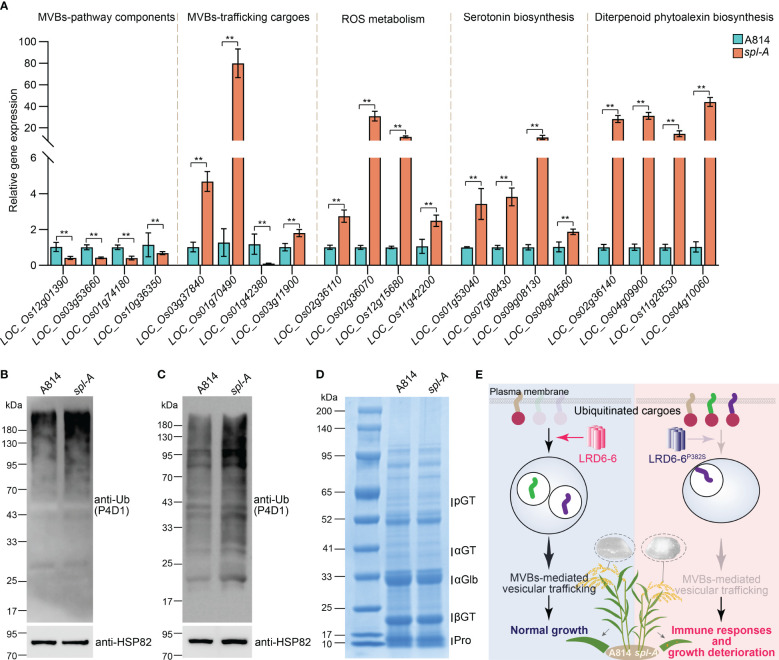
Characterization of the MVBs-mediated vesicle trafficking pathway in *spl-A*. **(A)** The mRNA transcriptional level of genes associated with MVBs-mediated vesicle trafficking pathway. The expression of genes that encoding possible MVBs-pathway components, possible MVBs-mediated trafficking cargoes, regulators involved in ROS metabolism, and key transcriptional factor and enzymes which regulate the biosynthesis of antimicrobial metabolites serotonin and diterpenoid phytoalexin was analyzed by RT-qPCR. The expression levels of the genes were normalized to the reference gene *Ubiquitin* and then normalized to the expression level of the genes in A814 (mean ± s.d., n = 3 technical repetitions). The detailed information of these genes was listed in **Supplementary Table S2**. All statistics were analyzed by Student’s *t*-test for *P* values (**, *P* <= 0.01). **(B)** Detection of the ubiquitinated conjugates in A814 and *spl-A* leaves. **(C)** Detection of the ubiquitinated conjugates in A814 and *spl-A* dry seeds. Total protein from A814 and *spl-A* leaves **(C)** or dry seeds **(C)** were respectively extracted and subjected to immunoblot analysis using the anti-Ub antibody (P4D1). The protein levels of HSP82 were detected to indicate the loading amount of total protein in each lane. **(D)** Comparison the protein profiles between A814 and *spl-A* dry seeds on SDS–PAGE gels stained by Coomassie blue. 20 μg of total protein for each sample was loaded. blue. pGT, 57-kDa proglutelins; αGT, 40-kDa glutelin acidic subunits; αGlb, 26-kDa a-globulin; βGT, 20-kDa glutelin basic subunits; Pro, prolamins. **(E)** An illustration summarizes how *spl-A* mutant activates immune responses and deteriorates plant growth in rice. The AAA ATPase LRD6-6 regulates the maturation of MVBs and promotes MVBs-mediated transportation of ubiquitinated proteins to guarantee normal growth of rice plant (left panel). The P382S mutation of LRD6-6 in *spl-A* impairs its ATPase activity and disrupts its interaction with the ESCRT-III subunits OsSNF7.1/7.2/7.3, thus compromise MVBs-mediated transportation process and leading to immune responses activation and growth deterioration including decreased rice quality (right panel).

To dissect the mechanism on how LRD6-6^P382S^ affects rice growth and immune responses, we then collected leaf samples from A814 and *spl-A* respectively before the emergence of the spotted lesions on *spl-A* and analyzed the mRNA transcriptional level of genes associated with MVBs-mediated vesicle trafficking pathway firstly (Zhu et al., 2016). We found genes encoding possible MVBs-pathway components and MVBs-trafficking cargoes were largely dysregulated in the *spl-A* mutant when compared to the wild-type A814 (**Figure 6A** and **Supplementary Table 3**), suggesting that the MVBs-mediated vesicle trafficking pathway is defective in *spl-A*. Deficient in MVBs-mediated vesicle trafficking will lead to ROS burst and antimicrobial metabolites accumulation (Zhu et al., 2016). We thus also detected the mRNA expression levels of the genes associated with these processes (Zhu et al., 2016). In accordance with previous report, the expression of genes involved in ROS metabolism and biosynthesis of antimicrobial metabolites serotonin and diterpenoid phytoalexin were found to increase significantly in *spl-A* (**Figure 6A** and **Supplementary Table 3**), suggesting *spl-A* may accumulate excess of ROS and antimicrobial metabolites which can activate immune responses and increase disease resistance.

The MVBs are well-known for their function in sorting ubiquitinated plasma membrane proteins (Hurley, 2008; Gao et al., 2014). To further characterize the effects of LRD6-6 P382S mutation in *spl-A*, we then detected the ubiquitination level of total protein from the *spl-A* mutant leaf. More ubiquitinated proteins were detected in *spl-A* by using the anti-Ub (P4D1) antibody when compared to A814 (**Figure 6B**), indicating P382S mutation of LRD6-6 leading to accumulate of more ubiquitinated proteins in plant. Together, our current results suggest that the enhanced immune responses and deteriorated growth of the *spl-A* mutant is likely caused by the compromise of MVBs-mediated vesicle trafficking which leading to ROS burst and antimicrobial metabolites accumulation.

Since *spl-A* exhibited increased chalky grain percentage and chalky grain degree (**Figures 1C–E**), we speculated that the MVBs-mediated vesicle trafficking of ubiquitinated proteins in seeds were also inhibited. Thus, we also explored the ubiquitination level of total protein from *spl-A* seeds. It was found that the ubiquitinated proteins accumulated in *spl-A* seeds were obviously more than that of A814 (**Figure 6C**), suggesting the MVBs-mediated vesicle trafficking is also inhibited in seed. The contents of storage proteins in rice seeds are important for establishment of grain quality including chalkiness (Li et al., 2022). We then compared the contents of major types of rice seeds storage proteins, glutelins, α-globulin and prolamins between *spl-A* and A814. The SDS-PAGE based Coomassie blue staining showed that there were no obvious differences in the content of storage proteins between *spl-A* and A814 seeds (**Figure 6D**). These results suggest that the compromised MVBs-mediated vesicle trafficking in *spl-A* seed deteriorate grain chalkiness likely through a pathway independent of storage proteins contents.

## Discussion

### The *spl-A* mutant exhibits activated immune responses and decreased grain quality in rice

The mutation of the *Spl-A* locus has been previously reported as *lesion mimic resembling* (*lmr*), *lesion resembling disease 6-6* (*lrd6-6*) and *spotted leaf 4* (*spl4*) in rice (Fekih et al., 2015; Zhu et al., 2016; Song et al., 2019). Like most of *spl* mutants, the *lmr* and *lrd6-6* mutants accumulated an excess of H_2_O_2_ and death cells, exhibited enhanced disease resistance to both fungal and bacterial diseases (Lorrain et al., 2003; Fekih et al., 2015; Zhu et al., 2016, 2020). Consistently with previous reports, the *spl-A* mutant also presented constitutive activated immune responses and enhanced broad-spectrum disease resistance (**Figure 2**, **Supplementary Figure 2** and **Supplementary Figure 3**). Besides immune responses, the *Spl-A* locus also regulates other aspects of plant phenotypes, such as leaf senescence and agronomic traits including grain weight, plant height and seed setting rate etc. It’s interesting that the *lrd6-6* and *spl-A* mutants exhibited decreased grain weight, while the *spl4* mutant showed increased grain weight (Fekih et al., 2015; Zhu et al., 2016; Song et al., 2019) (**Supplementary Figure 1E**). These results suggest that the *Spl-A* locus may regulate grain weight or even other traits depends on the genetic background of rice.

Although the *spl* mutants are broadly studied for their roles in immune responses, senescence, and plant growth or development (Zhu et al., 2020). It still remains unknown whether these mutants/genes regulate grain quality. In our study, we examined the chalkiness of the *spl-A* mutant and found that the *spl-A* mutant showed increased chalky grain percentage and chalky grain degree which deteriorates grain quality (**Figures 1C–E** and **Figure 3H–J**). Thus, our current study shed light on the new role of the *spl* mutants in grain quality regulation.

### The conserved 382^nd^ amino acid residue proline is essential for the full function of the AAA-type ATPase LRD6-6

The AAA-type ATPase family proteins contain conserved ATPase domains spanning 200–250 residues which cover the Walker A, Walker B and the SRH (Second Region of Homology) motifs that distinguish them from classic p-loop NTPases (Ogura and Wilkinson, 2001; Hanson and Whiteheart, 2005). VPS4 is a member of the AAA-type ATPase family which has been found to form hexamer and assemble with the ESCRT-III complex subunits to drive the maturation of MVBs (Babst et al., 1997; Schmidt and Teis, 2012). SKD1 and LRD6-6 are homologs of VPS4 from Arabidopsis and rice respectively, and they may function in MVBs maturation like that of VPS4 (Zhu et al., 2016). Several amino acid residues that essential for the function of VPS4/SKD1/LRD6-6 have been identified. For example, the 261^st^ (LRD6-6 as reference) lysine in Walker A motif is essential for nucleotide binding, the 315^th^ glutamic acid in Walker B is responsible for ATP hydrolysis, and the 372^nd^ arginine in SRH is vital for both ATP hydrolysis and oligomerization (Babst et al., 1998; Ogura et al., 2004; Zhu et al., 2016). The *spl-A* mutant in this study was caused by a single nucleotide mutation (C1144T) which led to a single amino acid substitution (P382S) in the AAA-type ATPase LRD6-6 (**Figures 3A–C**). Unlike the mutations in *lmr*, *lrd6-6* and *spl4* whose genes were destructed (Fekih et al., 2015; Zhu et al., 2016; Song et al., 2019), the mutation of *spl-A* provides us a unique allele to study the function of the 382^nd^ amino acid residue proline for LRD6-6.

The 382^nd^ amino acid residue proline locates in neither of the above-mentioned motifs of LRD6-6, but it’s conserved among this AAA-type ATPase family (**Figure 3D**). We then assayed the ATPase activity, the interaction with the ESCRT-III subunits OsSNF7.1/7.2/7.3 and OsVPS2, and the MVBs localization of the mutation protein LRD6-6^P382S^ from the *spl-A* mutant. Our results indicate that the P382S mutation of LRD6-6 impairs its ATPase activity and disrupts the interaction with the ESCRT-III subunits OsSNF7.1/7.2/7.3, while not affects its MVBs localization (**Figures 4**, **5** and **Supplementary Figures 4**, **5**). Thus, in this study, we have characterized a new conserved amino acid residue that is important for the full function of LRD6-6 and likely, for all the AAA-type ATPase family proteins since this site is conserved among them.

### LRD6-6 regulates MVBs-mediated vesicle trafficking to modulate rice immunity and grain quality

MVBs are single membrane bound organelles that serve as core converging station of the secretory and endocytic pathways for cargo trafficking in eukaryotic cells (Piper and Katzmann, 2007; Cui et al., 2016). Generally, ubiquitinated cargo proteins on the plasm membrane will be recognized and packaged into the intralumenal vesicles (ILVs) of MVBs by the ESCRT complexes. Then, these cargo proteins are transported by the MVBs to different destinations for proper functions (Piper and Katzmann, 2007; Cui et al., 2016; Zhu et al., 2023). Impairment of the MVBs-mediated vesicle trafficking pathway will lead to accumulate of ubiquitinated proteins in cells as reported in Yeast and Arabidopsis (Mizuno et al., 2003; Stringer and Piper, 2011; Gao et al., 2014; Katsiarimpa et al., 2014). However, whether rice cells will accumulate ubiquitinated proteins when this pathway is inhibited still remain unknown. In this study, we found ubiquitinated proteins accumulated in both the leaves and seeds of the *spl-A* mutant in which the MVBs-mediated vesicle trafficking was inhibited by mutation of the AAA-type ATPase LRD6-6 (**Figures 6B, C**). Thus, our discovery not only reveals the conserved roles of MVBs-mediated vesicle trafficking on the transportation of ubiquitinated proteins, but also expands its function in grain quality formation.

In summary, our study reveals that the conserved amino acid residue proline at the 382^th^ of the AAA-type ATPase LRD6-6 is essential for its ATPase activity and its interaction with the ESCRT-III subunits OsSNF7.1/7.2/7.3. Once this site is mutated, the MVBs-mediated vesicle trafficking pathway governed by LRD6-6 is compromised and rice plant harboring this mutation will accumulate excessive ubiquitinated cargo proteins in both leaves and seeds. Then, the immune responses of this mutant plant will be activated, and the growth and grain quality of this plant will be deteriorated (**Figure 6E**).

## Data availability statement

The original contributions presented in the study are included in the article/**Supplementary Material**. Further inquiries can be directed to the corresponding author/s.

## Author contributions

JY: Funding acquisition, Methodology, Resources, Writing – original draft, Data curation, Investigation, Validation. CZ: Investigation, Methodology, Validation, Writing – review & editing. QZ: Investigation, Methodology, Validation, Writing – review & editing. FL: Investigation, Methodology, Validation, Writing – review & editing. WH: Investigation, Formal analysis, Writing – review & editing. YiZ: Formal analysis, Investigation, Writing – review & editing. FM: Formal analysis, Investigation, Writing – review & editing. YuZ: Investigation, Writing – review & editing. BW: Investigation, Writing – review & editing. MZ: Investigation, Writing – review & editing. LZ: Formal analysis, Funding acquisition, Writing – review & editing. XZ: Formal analysis, Funding acquisition, Conceptualization, Methodology, Project administration, Resources, Supervision, Visualization, Writing – original draft, Writing – review & editing.

## Supplementary material

Supplementary Figure 1 Comparison on the main agronomic traits between and *spl-A*. The plant height **(A)**, tiller number **(B)**, panicle length **(C)**, seed setting rate **(D)** and 1,000-grain weight **(E)** of the *spl-A* mutant were respectively investigated and analyzed (mean ± s.d.). **(F–H)** Comparison on grain length **(G)** and grain width **(H)** between A814 and *spl-A* (mean ± s.d.). Representative photograph of ten grains were respectively shown. Bar = 1 cm. All statistics were analyzed by Student’s *t*-test for *P* values (**, *P* <= 0.01; ns, no significant differences).

Supplementary Figure 2 Evaluation the blast resistance of *spl-A* to different blast fungal isolates. Punch inoculation method was employed. The blast fungal isolates ZE-1 **(A–C)**, 0755-1-1 **(D–F)** and 99-20-2 **(G–I)** that are compatible with A814 was respectively used. Photograph of representative lesions **(A, D, G)** were shown. Lesion length **(B, E, H)** and the relative fungal growth **(C, F, I)** were measured at 5 day-post-inoculation (dpi). The relative fungal growth was determinated as fungi *MoPot2* DNA to rice *OsUbq* DNA by qPCR (mean ± s.d., n = 3 technical repetitions). Bars = 1 cm. All statistics were analyzed by Student’s *t*-test for *P* values (**, *P* <= 0.01).

Supplementary Figure 3 Evaluation the bacterial blight disease resistance of *spl-A* to different *Xoo* isolates. The resistance of *spl-A* to *Xoo* isolates P6 **(A–C)** and Xoo-4 **(D–F)** were tested. Photographs of representative leaves were taken at 14 dpi **(A, D)**. Disease lesion lengths **(B, E)** and bacterial populations **(C, F)** of A814 and the *spl-A* mutant were measured at 0, 7 and 14 dpi respectively (mean ± s.d., n = 12 for lesion lengths and n = 3 for bacterial populations). Bars = 1 cm. All statistics were analyzed by Student’s *t*-test for *P* values (**, *P* <= 0.01).

Supplementary Figure 4 Detection the self-interaction of LRD6-6^P382S^ protein. The self-interaction of LRD6-6^P382S^ was detected by using both Y2H **(A)** and BiFC **(B)** approaches. BD, pGBKT7; AD, pGADT7; DDO, double dropout medium ((SD/–Leu/–Trp); QDO/A, quadruple dropout medium supplemented with Aureobasidin A (SD/–Ade/–His/–Leu/–Trp/AbA). BiFC assay was performed in *N. benthamiana*. The green fluorescence signals present the interaction between the tested proteins, the red signals represent the auto-fluorescence of chlorophyll. Some of the green fluorescence signals are indicated by white arrows to clearly shown the interaction. Bar = 20 μm.

Supplementary Figure 5 Subcellular localization of the protein LRD6-6^P382S^. **(A)** Determination the subcellular localization of protein LRD6-6^P382S^ fused with green fluorescence protein (LRD6-6^P382S^-GFP) in *N. benthamiana*. The RabF1/ARA6 protein that fused with red fluorescence protein (RabF1/ARA6-RFP) was used as the multivesicular bodies (MVBs) marker. Some of the overlapped punctate fluorescence signals were indicated by white arrows to clearly shown the co-localization. The fluorescence intensity along the white line was also measured and shown to indicate the co-localization **(B)**. The co-localization of the wild-type LRD6-6-GFP with RabF1/ARA6-RFP was included as control **(C, D)**. Bar = 20 μm.
