# Supplementary figures and images for: A single amino acid substitution in the AAA-type ATPase LRD6-6 activates immune responses but decreases grain quality in rice
Source: Front Plant Sci. 2024 Aug 6;15:1451897. doi: 10.3389/fpls.2024.1451897 (PMC11333209; doi:10.3389/fpls.2024.1451897)

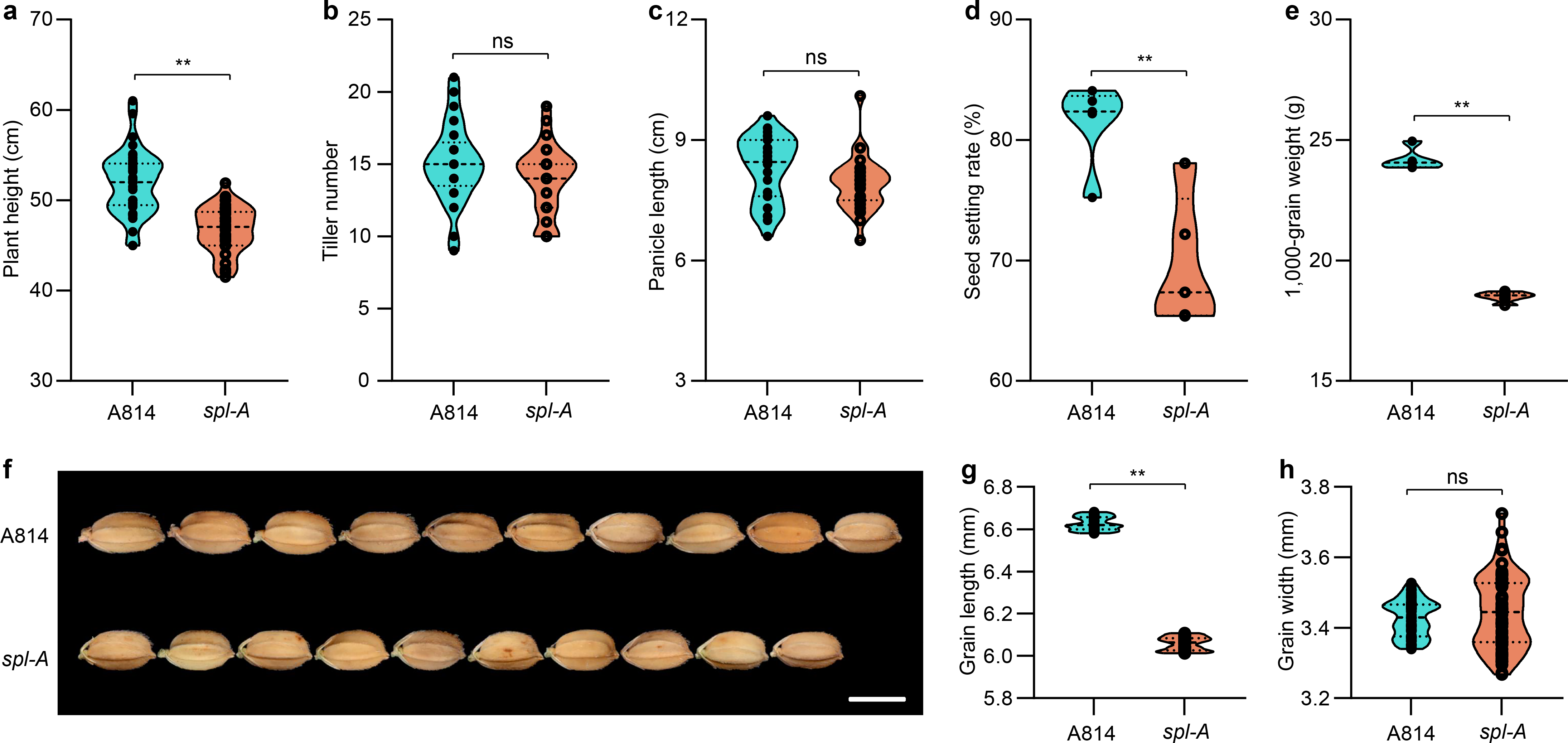

Supplement: Supplementary Figure 1 — Comparison on the main agronomic traits between and spl-A. The plant height (A), tiller number (B), panicle length (C), seed setting rate (D) and 1,000-grain weight (E) of the spl-A mutant were respectively investigated and analyzed (mean ± s.d.). (F–H) Comparison on grain length (G) and grain width (H) between A814 and spl-A (mean ± s.d.). Representative photograph of ten grains were respectively shown. Bar = 1 cm. All statistics were analyzed by Student’s t-test for P values (**, P <= 0.01; ns, no significant differences). [file Image_1.tif]

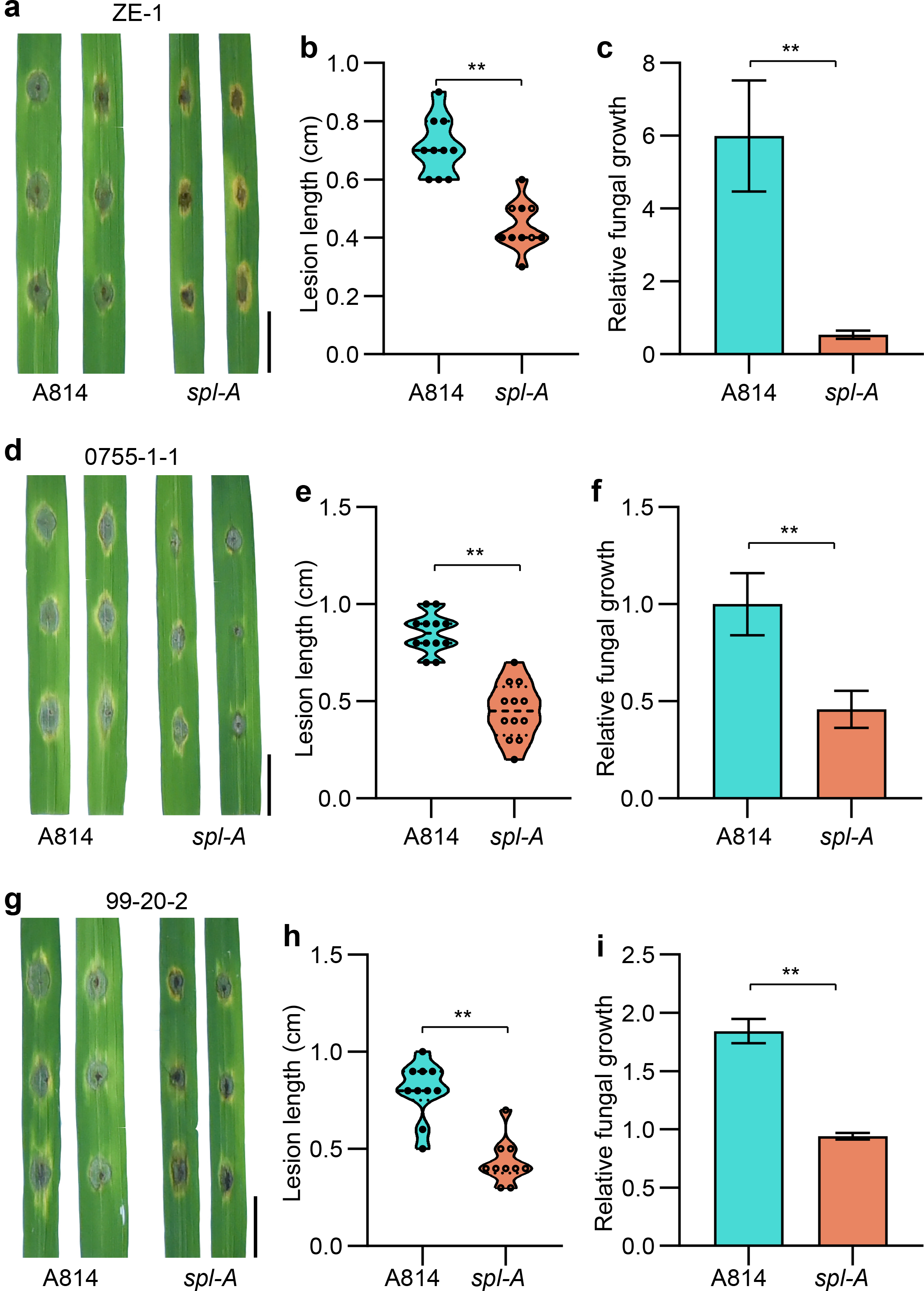

Supplement: Supplementary Figure 2 — Evaluation the blast resistance of spl-A to different blast fungal isolates. Punch inoculation method was employed. The blast fungal isolates ZE-1 (A–C), 0755-1-1 (D–F) and 99-20-2 (G–I) that are compatible with A814 was respectively used. Photograph of representative lesions (A, D, G) were shown. Lesion length (B, E, H) and the relative fungal growth (C, F, I) were measured at 5 day-post-inoculation (dpi). The relative fungal growth was determinated as fungi MoPot2 DNA to rice OsUbq DNA by qPCR (mean ± s.d., n = 3 technical repetitions). Bars = 1 cm. All statistics were analyzed by Student’s t-test for P values (**, P <= 0.01). [file Image_2.tif]

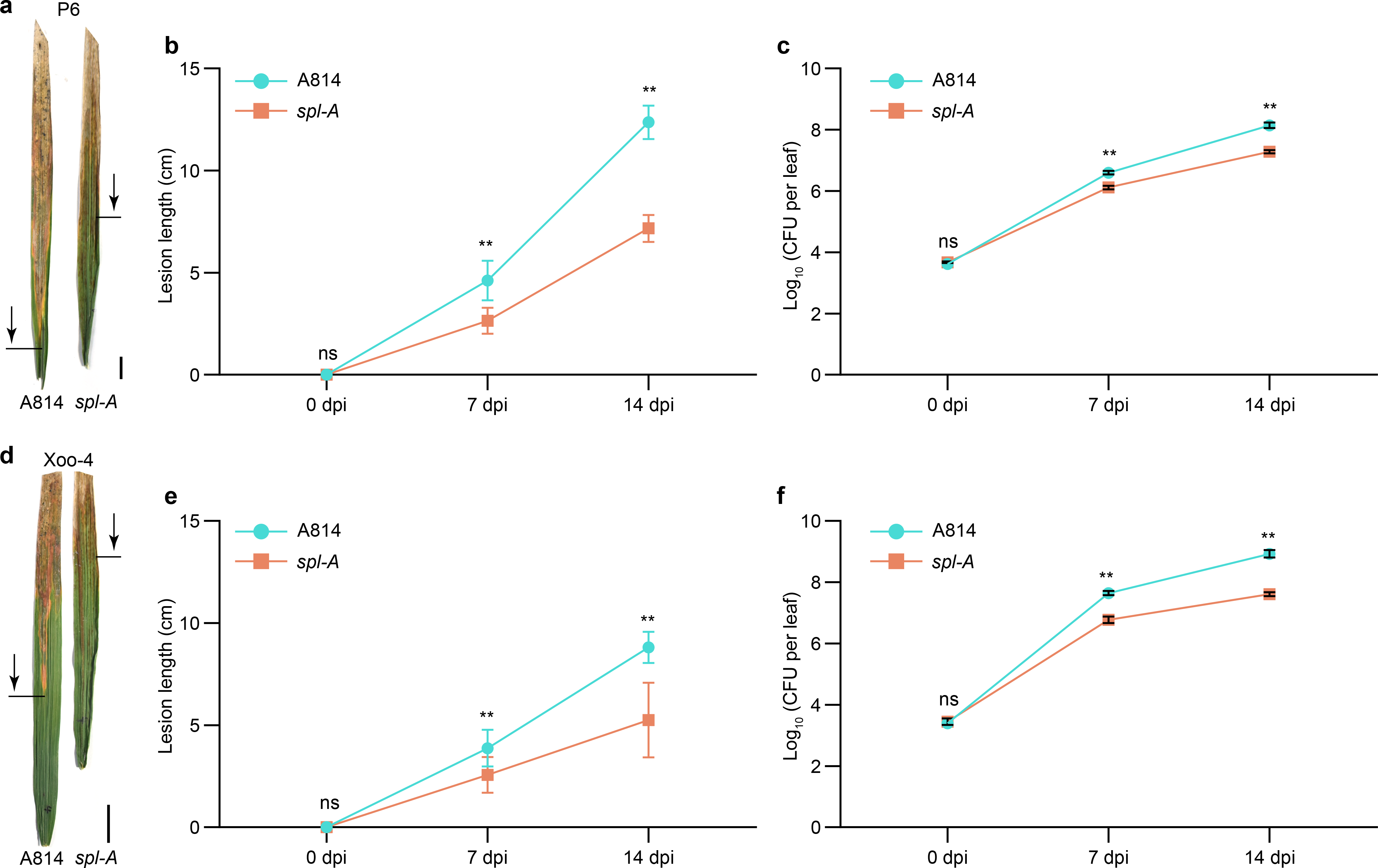

Supplement: Supplementary Figure 3 — Evaluation the bacterial blight disease resistance of spl-A to different Xoo isolates. The resistance of spl-A to Xoo isolates P6 (A–C) and Xoo-4 (D–F) were tested. Photographs of representative leaves were taken at 14 dpi (A, D). Disease lesion lengths (B, E) and bacterial populations (C, F) of A814 and the spl-A mutant were measured at 0, 7 and 14 dpi respectively (mean ± s.d., n = 12 for lesion lengths and n = 3 for bacterial populations). Bars = 1 cm. All statistics were analyzed by Student’s t-test for P values (**, P <= 0.01). [file Image_3.tif]

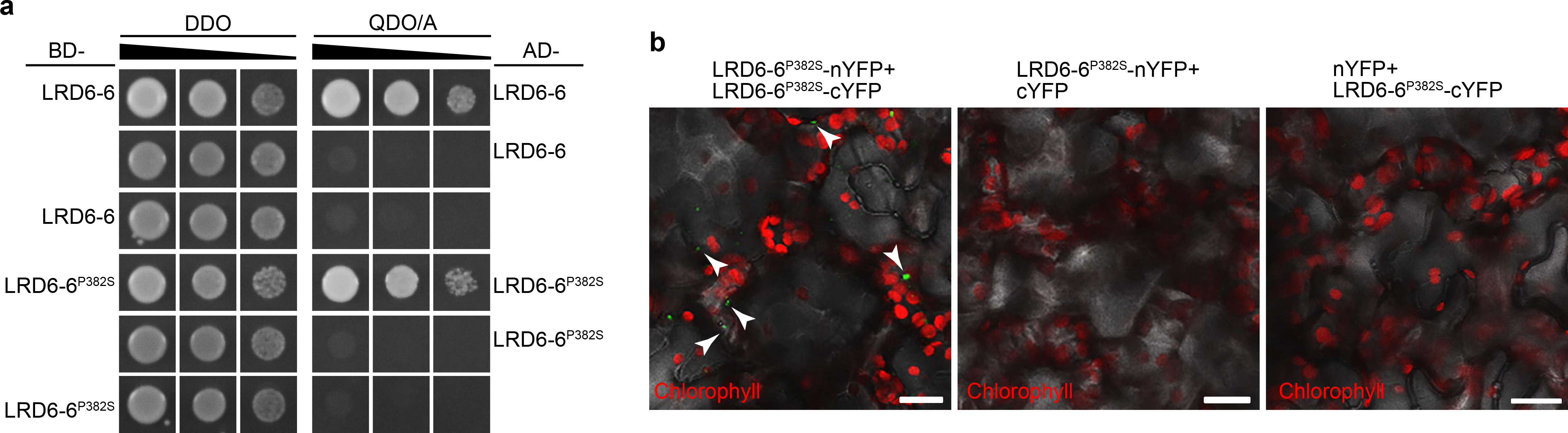

Supplement: Supplementary Figure 4 — Detection the self-interaction of LRD6-6P382S protein. The self-interaction of LRD6-6P382S was detected by using both Y2H (A) and BiFC (B) approaches. BD, pGBKT7; AD, pGADT7; DDO, double dropout medium ((SD/–Leu/–Trp); QDO/A, quadruple dropout medium supplemented with Aureobasidin A (SD/–Ade/–His/–Leu/–Trp/AbA). BiFC assay was performed in N. benthamiana. The green fluorescence signals present the interaction between the tested proteins, the red signals represent the auto-fluorescence of chlorophyll. Some of the green fluorescence signals are indicated by white arrows to clearly shown the interaction. Bar = 20 μm. [file Image_4.tif]

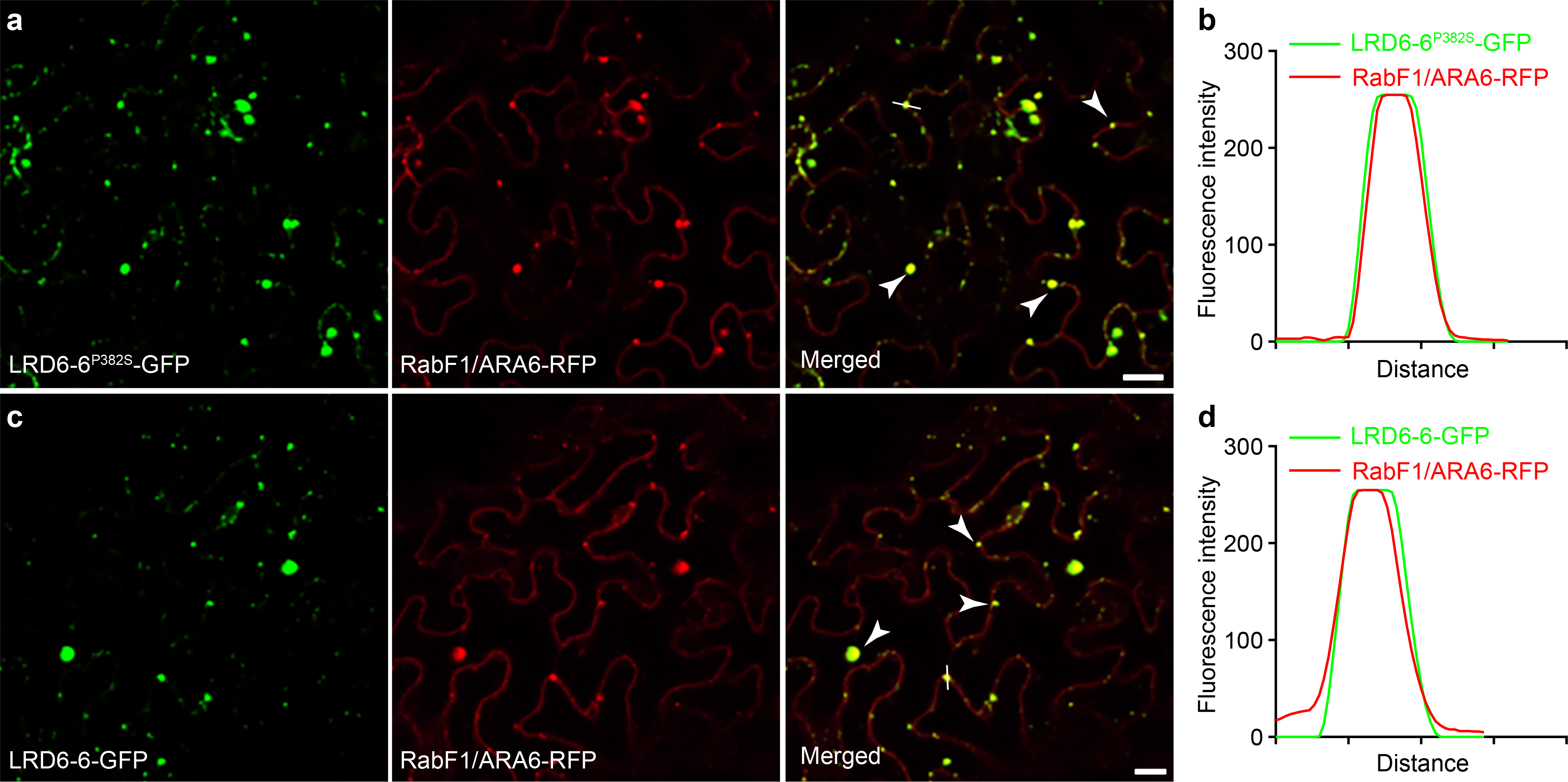

Supplement: Supplementary Figure 5 — Subcellular localization of the protein LRD6-6P382S. (A) Determination the subcellular localization of protein LRD6-6P382S fused with green fluorescence protein (LRD6-6P382S-GFP) in N. benthamiana. The RabF1/ARA6 protein that fused with red fluorescence protein (RabF1/ARA6-RFP) was used as the multivesicular bodies (MVBs) marker. Some of the overlapped punctate fluorescence signals were indicated by white arrows to clearly shown the co-localization. The fluorescence intensity along the white line was also measured and shown to indicate the co-localization (B). The co-localization of the wild-type LRD6-6-GFP with RabF1/ARA6-RFP was included as control (C, D). Bar = 20 μm. [file Image_5.tif]
