# Supplementary material for: A single amino acid substitution in the AAA-type ATPase LRD6-6 activates immune responses but decreases grain quality in rice
Source: Front Plant Sci. 2024 Aug 6;15:1451897. doi: 10.3389/fpls.2024.1451897 (PMC11333209; doi:10.3389/fpls.2024.1451897)
Supplement: Supplementary file 7 [file Table_2.docx]

**Supplementary Table 2.** Genetic analyses of the *Spl-A* locus.

| **Cross** | **F_1_ phenotype** | **F_2_ population** | | **Total** | **χ2 (3:1,0.05)=3.84** |
| --- | --- | --- | --- | --- | --- |
|  |  | Wide type | Mutant type |  |  |
| *spl-A*×Jodan | Wide type | 378 | 112 | 490 | 1.088 |
| Jodan×*spl-A* | Wide type | 383 | 107 | 490 | 2.449 |
