# Supplementary material for: A single amino acid substitution in the AAA-type ATPase LRD6-6 activates immune responses but decreases grain quality in rice
Source: Front Plant Sci. 2024 Aug 6;15:1451897. doi: 10.3389/fpls.2024.1451897 (PMC11333209; doi:10.3389/fpls.2024.1451897)
Supplement: Supplementary file 8 [file Table_3.docx]

**Supplementary Table 3.** List of genes associated with MVBs-mediated vesicle trafficking analyzed in this study.

| **Classification** | **Gene ID** | **Annotation** |
| --- | --- | --- |
| MVBs-pathway components | *LOC_Os12g01390* | Clathrin heavy chain |
|  | *LOC_Os03g53660* | OsMyoXIE-1 |
|  | *LOC_Os01g74180* | Adaptin |
|  | *LOC_Os10g36350* | MSP domain containing protein |
| MVBs-trafficking cargoes | *LOC_Os03g37840* | OsHAK16 |
|  | *LOC_Os01g70490* | OsHAK5 |
|  | *LOC_Os01g42380* | OsPDR9 |
|  | *LOC_Os03g11900* | OsMST4 |
| ROS metabolism | *LOC_Os02g36110* | CYP76M7 |
|  | *LOC_Os02g36070* | CYP76M8 |
|  | *LOC_Os12g15680* | Laccase precursor protein |
|  | *LOC_Os11g42200* | Laccase precursor protein |
| Serotonin biosynthesis | *LOC_Os01g53040* | OsWRKY14 |
|  | *LOC_Os07g08430* | TS |
|  | *LOC_Os09g08130* | IGPS |
|  | *LOC_Os08g04560* | OsTDC3 |
| Diterpenoid phytoalexin biosynthesis | *LOC_Os02g36140* | OsDTC1/OsKSL7 |
|  | *LOC_Os04g09900* | OsCPS4/OsCyc1 |
|  | *LOC_Os11g28530* | OsDTC2/OsKSL8 |
|  | *LOC_Os04g10060* | OsKSL4/OsDTS2 |

* These genes have been summarized and used in a previous study (Zhu et al., 2016). The primers used for RT-qPCR analysis were also designed by the previous study.

**Reference**

Zhu, X., Yin, J., Liang, S., Liang, R., Zhou, X., Chen, Z., Zhao, W., Wang, J., Li, W., He, M., Yuan, C., Miyamoto, K., Ma, B., Wang, J., Qin, P., Chen, W., Wang, Y., Wang, W., Wu, X., Yamane, H., Zhu, L., Li, S., and Chen, X. (2016). The multivesicular bodies (MVBs)-localized AAA ATPase LRD6-6 inhibits immunity and cell death likely through regulating MVBs-mediated vesicular trafficking in rice. *PLOS Genetics* 12, e1006311. doi:10.1371/journal.pgen.1006311.
